# Review and critique of current testing protocols for upper-limb prostheses: a call for standardization amidst rapid technological advancements

**DOI:** 10.3389/frobt.2023.1292632

**Published:** 2023-11-15

**Authors:** Joshua R. Siegel, Marcus A. Battraw, Eden J. Winslow, Michelle A. James, Wilsaan M. Joiner, Jonathon S. Schofield

**Affiliations:** ^1^ Department of Mechanical and Aerospace Engineering, University of California, Davis, Davis, CA, United States; ^2^ Department of Biomedical Engineering, University of California, Davis, Davis, CA, United States; ^3^ Shriners Hospital for Children, Northern California, Sacramento, Sacramento, CA, United States; ^4^ Department of Neurobiology, Physiology and Behavior, University of California, Davis, Davis, CA, United States; ^5^ Department of Neurology, University of California, Davis, Davis, CA, United States

**Keywords:** prosthesis evaluation, upper-limb robotic prostheses, validated task-based assessments, dexterity assessment, control system assessment, robotic prosthesis performance

## Abstract

This article provides a comprehensive narrative review of physical task-based assessments used to evaluate the multi-grasp dexterity and functional impact of varying control systems in pediatric and adult upper-limb prostheses. Our search returned 1,442 research articles from online databases, of which 25 tests—selected for their scientific rigor, evaluation metrics, and psychometric properties—met our review criteria. We observed that despite significant advancements in the mechatronics of upper-limb prostheses, these 25 assessments are the only validated evaluation methods that have emerged since the first measure in 1948. This not only underscores the lack of a consistently updated, standardized assessment protocol for new innovations, but also reveals an unsettling trend: as technology outpaces standardized evaluation measures, developers will often support their novel devices through custom, study-specific tests. These boutique assessments can potentially introduce bias and jeopardize validity. Furthermore, our analysis revealed that current validated evaluation methods often overlook the influence of competing interests on test success. Clinical settings and research laboratories differ in their time constraints, access to specialized equipment, and testing objectives, all of which significantly influence assessment selection and consistent use. Therefore, we propose a dual testing approach to address the varied demands of these distinct environments. Additionally, we found that almost all existing task-based assessments lack an integrated mechanism for collecting patient feedback, which we assert is essential for a holistic evaluation of upper-limb prostheses. Our review underscores the pressing need for a standardized evaluation protocol capable of objectively assessing the rapidly advancing prosthetic technologies across all testing domains.

## 1 Introduction

Standardized, reliable, and validated task-based evaluation measures for upper-limb prostheses are crucial for advancing research and, most importantly, enhancing patient care. Using a task-based approach, which entails manipulating physical objects with prostheses, presents a distinct advantage as it directly assesses a patient’s performance in real-time. While self-reported surveys are invaluable in detailing patient functional outcomes, task-based methods can provide unique and complementary information while also helping to mitigate challenges with these approaches. Notably, surveys often encounter biases such as: recall bias (participants might not accurately remember their experiences), social desirability bias (participants might answer in a way to be viewed favorably by others), and extreme response bias (participants might tend to choose the highest or lowest score on a rating scale) (Choi and Pak, 2004).

Task-based measures not only play a pivotal role in examining performance but can also inform clinical decision-making, potentially guiding clinicians in choosing the optimal prosthetic device or strategy tailored to individual patients. First, employing the right evaluation measure allows for precise tracking of patient progress, evidencing the effectiveness of treatments or indicating necessary adjustments. Second, these measures provide objective data to substantiate cost justifications for insurance and public health systems, facilitating transparency among stakeholders (including researchers, clinicians, patients, insurance agencies, regulatory bodies, and the general public). Finally, the standardization of task-based measures ensures consistent comparisons across different prosthetic devices and control systems. This objective data is essential for iterative improvement and innovation, helping reduce uncertainties introduced by study-specific measurement techniques. In essence, standardized evaluation measures, when adeptly implemented, are the cornerstone for advancements in upper-limb prosthetics.

**FIGURE 1:**
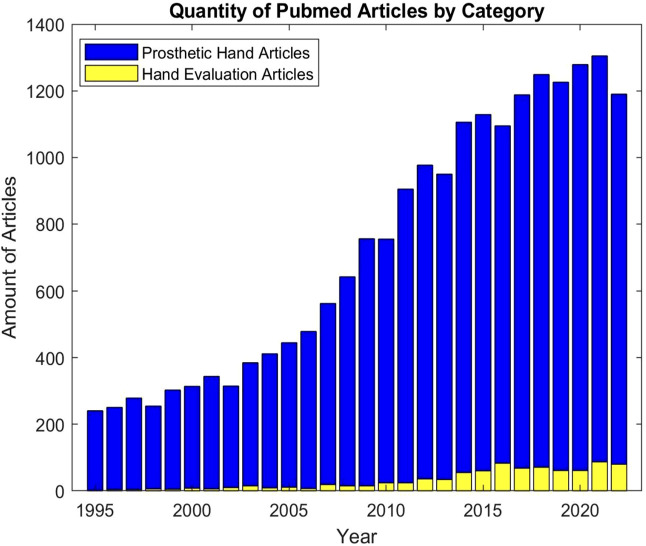
Quantity of pubmed articles by category.

However, despite the clear significance of these measures, there is a notable discrepancy in the focus of upper-limb prosthetic research. While the mechatronics of upper-limb prostheses has seen tremendous progress, a standardized and well adopted assessment framework still remains absent (Vujaklija et al., 2016). This has caused researchers to resort to creating boutique tests for new features. To illustrate this point, we conducted a search on PubMed for articles discussing new upper-limb prosthetic technology *versus* those discussing testing methodologies. For new prosthetic technologies, we used the keywords: ‘Prosthetic Hand’, ‘New Upper-Limb Prosthesis’, and ‘Hand Neuroprosthesis’. We used ‘prosthetic hand test’, ‘prosthetic hand dexterity assessment’, ‘prosthetic control system test’, and ‘prosthesis control system assessment’ for evaluation methods. As depicted in Figure 1, the results of this search yield a stark discrepancy between the substantial volume of scientific literature reporting on upper-limb prosthetic technologies and the limited number of articles reporting on techniques to evaluate these same devices.

### 1.1 Review scope

The scope of this paper is to analyze task-based assessments that evaluate both dexterity (the precise, voluntary movements required when handling objects), and the functional impact of varying control systems (the technology {electrical, mechanical, or other} that interfaces with the user to actuate a prosthetic device) (Backman et al., 1992). Our novel approach contrasts prior upper-limb prosthetic assessment reviews by Yancosek et al., in 2009, Resnik et al., in 2017, and Wang et al., in 2018 which utilized different evaluation criteria, focused solely on dexterity evaluations, and included surveys along with assessments for stroke-patients (Yancosek and Howell, 2009; Resnik et al., 2017a; Wang et al., 2018). Conversely, we exclusively examined task-based assessments for the multi-grasp dexterity and functional impact of varying control systems in pediatric and adult upper-limb prostheses. For our evaluation, we used a diverse set of criteria, emphasizing accuracy, performance, reliability, and validity (Cornell, 2023; Opentext, 2023). Additionally, we understand the importance of assessing patient performance both with and without a prosthesis, particularly in bimanual tasks. However, to keep our focus on evaluating the functionality of a prosthesis or a patient’s ability to use it, this metric was omitted from our evaluation criteria but will be noted in the descriptions of relevant tests, if applicable. We also acknowledge the growing body of literature reporting on prosthesis interfaces that restore sensory feedback to users. Sensory feedback is poised to be an integral component of future prostheses and thus, there is a growing body of literature describing novel assessments which merits its own review (Markovic et al., 2018; Beckler et al., 2019; Williams et al., 2019; Marasco et al., 2021; Battraw et al., 2022; Cheng et al., 2023). Our review is exclusively analyzing functional tasks sensitive to changes in patient motor-function without the requisite inclusion of a sensory feedback system. Thus, we excluded tests that are designed specifically to evaluate sensory enabled upper-limb prosthetic systems.

## 2 Methods

**FIGURE 2:**
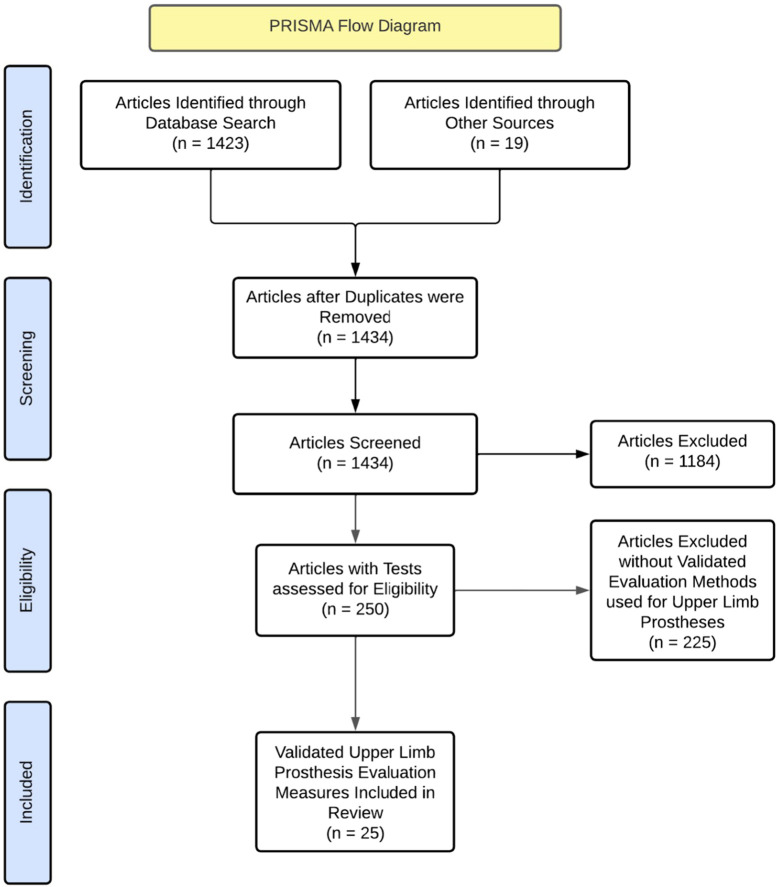
Prisma flow diagram for test selection.

Our review consisted of 1,423 journal articles sourced from online databases: PubMed, Medline, Shirley-Ryan Rehabilitation Measures, Google Scholar, and ScienceDirect. Search terms for each database included: journal article [Publication Type] AND ‘prosthetic hand test’ OR ‘prosthetic hand dexterity assessment’ OR ‘prosthetic control system test' OR ‘prosthesis control system assessment’) and 19 articles from previous knowledge. After duplicates were removed, we were left with 1,434 articles. After reading titles and abstracts, we narrowed our scope to 250 papers. From these, we selected articles that presented a validated task for measuring either upper-limb prosthetic dexterity, control systems, or both. The task could include a questionnaire but articles that solely consisted of questionnaires were excluded. After examination, 25 tests were identified for inclusion in our study. This selection process has been shown in Figure 2.

### 2.1 Assessment criteria

Our criteria, as described below, were designed to measure the effectiveness of tests across a broad range of factors which we grouped into two categories [1] Dexterity and Control Systems Criteria, and [2] Additional Considerations. A summary of our criteria is provided in Table 1.

**TABLE 1:** Custom evaluation criteria.

Criteria	Assessment scoring
**Holistic Assessment**	Did the evaluation test both dexterity and control systems, including multiple hand grasps?
**Object Size**	Did the test use varying sized objects?
**Object Compliance**	Did the test use objects that had varying compliances/densities?
**In-Hand Manipulation**	Did the test require the participant to move objects within the prosthetic hand?
**Tool Usage**	Did the test include utilizing tools?
**Monitoring Progress**	Does the test or associated research report its capability to effectively monitor patient progress?
**Patient Feedback**	Did the test provide a method to include patient feedback, such as a questionnaire?
**Evaluator Expertise**	Did the test require the evaluator to have prior training or undergo test-specific training?

#### 2.1.1 Dexterity and Control Systems Criteria

We first assessed whether the test offered a *holistic assessment* by analyzing both dexterity (defined as inclusion of at least five common hand grasps) and control systems (defined as the test’s ability to be sensitive in detecting performance variations due to different control systems) (Vergara et al., 2014). Next, we evaluated whether the manipulated test objects require the prosthesis to perform a range of grasping movements with varying degrees of hand closure and force, mirroring real-world applications. Specifically, we considered *object size* and *object compliance*. Our evaluation process also included *in-hand manipulation* capabilities, as the prosthesis’s ability to securely hold and manipulate objects signifies its capacity for complex movements beyond a simple grasp. *Tool usage* was our final dexterity and control systems criterion since manipulating tools, such as screwdrivers or toothbrushes, are integral to everyday life.

#### 2.1.2 Additional Considerations

*Monitoring progress* was also a central aspect of our evaluation, as gauging the test’s ability to effectively track changes in dexterity or control systems is essential for assessing efficacy or indicating the need for adjustments. To evaluate this, we identified whether the test or associated research reported its capability to effectively monitor progress. We also highly value the inclusion of *patient feedback*, captured through questionnaires or other methods, as it provides a user-centric perspective on the prosthesis’s performance. Our criteria also accounted for the *evaluators' expertise* requirements; some examples include backgrounds in Physical Therapy, Occupational Therapy, or Engineering. *Efficiency* of test administration and *accessibility* also factored into our evaluation process since balancing time-constraints, availability, and affordability with quality insights is essential to widespread use of comprehensive evaluations.

#### 2.1.3 Reliability metrics

In addition to our criteria, we also assessed the reliability of each measure. Reliability is a fundamental psychometric property that pertains to the consistency of a measure or test over time (Backman et al., 1992; Opentext, 2023). When the same test is administered to the same individual or group under identical conditions, a reliable test should yield the same or very similar results (Backman et al., 1992; Opentext, 2023). Our evaluation included three types of reliability: test-retest, inter-rater, and internal consistency. Test-retest reliability refers to the stability of a test over time, meaning if the same test is given to the same participants multiple times, the results should be very similar or identical (Opentext, 2023). Inter-rater reliability evaluates the extent of agreement among multiple raters or observers (Opentext, 2023). This is crucial when human observers are involved in data collection to mitigate the risk of subjectivity or bias (Opentext, 2023). Internal consistency gauges the stability of results across items within a test (Opentext, 2023). High internal consistency suggests that the items of the test are likely measuring the same underlying construct (Opentext, 2023). By examining these three dimensions of reliability, we achieved a comprehensive and robust assessment of each measure’s dependability.

To evaluate the test-retest and inter-rater reliability of each assessment, we used reported Intraclass Correlation Coefficient (ICC) values (Bravo and Potvin, 1991). ICC values range from 0 to 1, with values near 1 indicating high reliability, while those close to 0 suggest low reliability (Bravo and Potvin, 1991). If internal consistency was applicable, we referenced the reported Cronbach’s alpha (α) values (Bravo and Potvin, 1991). Cronbach’s alpha values also range from 0 to 1; values near 1 indicate high internal consistency, and those close to 0 indicate low internal consistency (Bravo and Potvin, 1991). Our scoring range for ICC and α are shown in Table 2 (Bravo and Potvin, 1991).

**TABLE 2:** Reliability scoring.

Reliability measure	≥0.90	0.90>ICC ≥ 0.7	<0.7	Not reported
**ICC**	Excellent	Good	Poor	✖
**α**	Excellent	Good	Poor	✖

#### 2.1.4 Validation metrics

Another essential psychometric property for evaluating tests of prosthetic hand dexterity and control systems is validation. Validity refers to the degree to which the test accurately and reliably measures what it intends to, ensuring the inferences and conclusions drawn from the test results are appropriate and meaningful (Backman et al., 1992; Cornell, 2023; Opentext, 2023). Furthermore, it is critical to incorporate various types of validity; we included face, content, construct, external, concurrent, and predictive (Backman et al., 1992; Cornell, 2023; Opentext, 2023). Face validity ensures that the test appears to be measuring what is intended (Cornell, 2023; Opentext, 2023). Content validity guarantees a comprehensive measurement of all facets of the subject (Cornell, 2023; Opentext, 2023). Construct validity, on the other hand, ensures that the designed measurement tool accurately assesses what it purports to measure (Cornell, 2023; Opentext, 2023). External validity confirms the generalizability of the test results to real-world scenarios or contexts beyond the experimental environment (Cornell, 2023; Opentext, 2023). Concurrent validity involves a comparison with an existing test or established criterion to ascertain the validity of the new test (Cornell, 2023; Opentext, 2023). Lastly, predictive validity establishes the link between test scores and performance in a specific domain, aiding in the prediction of future performance based on current assessments (Cornell, 2023; Opentext, 2023). Each layer of validity was crucial in providing a comprehensive and effective evaluation of prosthetic hand dexterity and control system tests. Our validity scoring criteria are shown in Table 3 and were adapted from Resnik et al. (Resnik et al., 2017a).

**TABLE 3:** Validity criteria.

Validity type	Excellent	Adequate	Poor	No evidence
**Face**	Test clearly appears to measure what is intended	Test appears to measure what is intended	Test does not appear to measure what is intended	No Evidence Available
**Content**	Test evaluates both dexterity with multiple hand grasps and control system	Test evaluates either dexterity with multiple hand grasps or control system	Test only evaluates a single hand grasp or control system	No Evidence Available
**Construct**	Results of the test match all the intended measures	Results of the test match some of the intended measures	Results of the test do not match the intended measures	No Evidence Available
**External**	The test uses objects and tasks applicable to daily life	The test uses some objects and tasks applicable to daily life	The test does not use objects and tasks applicable to daily life	No Evidence Available
**Concurrent**	The test has been compared with multiple other validated tests	The test has been compared with one other validated test	The test has not been compared with other validated tests	No Evidence Available
**Predictive**	The test accounts for future improvements in prostheses	The test can measure current state-of-the-art prostheses	The test is incapable of measuring current state-of-the-art prostheses	No Evidence Available

## 3 Results

We have provided a brief description for each of the 25 tests below, discussing them within the context of our evaluation criteria and their psychometric properties. For context and comparison, the median administration time across all tests is ∼25 min and the median cost per test is ∼$200. The results of these evaluations have been summarized in Tables 4-7.

**TABLE 4:** Dexterity and control system scoring.

Test	Holistic assessment	Object size	Object compliance	In-hand manipulation	Tool usage
**ACMC**	DM	SML	MC	✔	✔
**AHAP**	DM	SML	MC	✔	✖
**AM-ULA**	DCM	SML	MC	✔	✔
**AOSDT**	DM	SM	SC	✔	✔
**ARAT**	DM	SM	CO	✔	✖
**BAM-ULA**	DM	SML	SC	✔	✔
**BBT**	DO	S	CO	✖	✖
**CAPPFUL**	DCM	SML	SC	✔	✔
**CQT**	DCM	SM	CO	✔	✔
**ECB**	DM	SML	SC	✔	✖
**FDT**	DO	S	CO	✔	✖
**GaMA**	DCM	M	SC	✖	✖
**JHFT**	DM	SM	SC	✔	✔
**MMDT**	DO	S	CO	✔	✖
**NHPT**	DO	S	CO	✔	✖
**PHAM**	DCM	SM	CO	✔	✖
**PPT**	DO	S	CO	✔	✖
**RCRT**	DO	S	CO	✔	✖
**SHAP**	DCM	SM	SC	✔	✔
**SHFT**	DM	SML	SC	✔	✔
**SODA**	DM	SM	SC	✔	✖
**T-MAP**	DCM	SML	MC	✔	✔
**UBET**	DCM	SML	SC	✔	✔
**UNBT**	DCM	SML	SC-MC****	✔	✔
**WMFT**	DM	SM	SC	✔	✖

Key: DO, dexterity only; DM, dexterity only but uses multiple hand grasps; DC, dexterity and control system; DCM, dexterity and control system and uses multiple hand grasps; MC, multiple custom objects; MH, multiple household objects; SO, single object, S = Small* Objects Only, M = medium objects only, SM, Small and Medium** Objects, SML, small, Medium, and Large*** Objects, MC, Multiple Compliances ( ≥ 5), SC, Some Compliances (5>SC ≥ 2), CO, single compliance only.

^a^ A small object can be moveable using only fingers on an average size able-body participant.

^b^ A medium object can be held mostly within the hand of an average size able-body participant.

^c^ A large object will have an external component outside of the hand (such as a frying pan) or requires both hands (such as a tire).

^d^ Depends on the subset used.

**TABLE 5:** Additional considerations scoring.

Test	Monitoring progress	Patient feedback	Evaluator Expertise	Efficient administration	Accessibility considerations	Year
**ACMC**	✔	✖	NQ	>30 min	∼$1,000, P	2008
**AHAP**	✖	✖	NQ	>30 min	∼$200, P	2019
**AM-ULA**	✔	✖	CHT	>30 min	∼$200, P	2012
**AOSDT**	✖	✖	NQ	>30 min	∼$250, C	2022
**ARAT**	✔	✖	CHT	<30 min	∼$650, P	1981
**BAM-ULA**	✔	✖	NQ	<15 min	∼$100, P	2017
**BBT**	✔	✖	NQ	<5 min	∼$200, P	1985
**CAPPFUL**	✔	✔	OT	>25 min	∼$300, P	2018
**CQT**	✔	✖	NQ	>15 min	∼$200, P	1965
**ECB**	✖	✖	NQ	>30 min	∼$200, P	2020
**FDT**	✔	✖	NQ	<5 min	∼$90, P	2003
**GaMA**	✔	✖	R	>1 h	>$2000, P	2018
**JHFT**	✔	✖	NQ	<25 min	∼$400, P	1969
**MMDT**	✔	✖	NQ	<10 min	∼$350, P	1997
**NHPT**	✔	✖	NQ	<5 min	∼$100, P	1985
**PHAM**	✔	✖	R	>1 h	>$500, P/C	2017
**PPT**	✔	✖	NQ	<5 min	∼$150, P	1948
**RCRT**	✔	✖	NQ	<5 min	∼120, P	2016
**SHAP**	✔	✖	NQ	>45 min	>$2,500, P	2002
**SHFT**	✔	✖	NQ	<25 min	∼$100, P	1995
**SODA**	✔	✖	NQ	>30 min	∼$100, P	1996
**T-MAP**	✔	✖	NQ	>10 min	∼$50, P	2017
**UBET**	✔	✖	OT	>30 min	∼$400, P	2006
**UNBT**	✔	✖	NQ	>30 min	∼$500, P	1985
**WMFT**	✔	✖	NQ	>30 min	∼$100, P	2001

Key: CHT, certified hand therapist; OT, occupational therapist, R = researcher, NQ, no required qualifications, C = custom made, P = Purchasable/commonly available.

**TABLE 6:** Reliability scoring.

Test	Test-retest	Inter-rater	Internal consistency
**ACMC**	ICC: 0.94	ICC: 0.92 to 0.95	✖
**AHAP**	ICC: 0.839	ICC: 0.969	α: 0.846
**AM-ULA**	ICC: 0.91	ICC: 0.69 to 0.95	α: 0.89 to 0.91
**AOSDT**	✖	✖	✖
**ARAT**	ICC: 0.965	ICC: 0.998	α: 0.985^a^
**BAM-ULA**	ICC: 0.91	✖	α: 0.83
**BBT**	ICC: 0.96	ICC: 0.99	✖
**CAPPFUL**	✖	ICC: 0.88 to 0.99	α: 0.79 to 0.82
**CQT**	✖	ICC: 0.92 to 1^a^	✖
**ECB**	✖	✖	✖
**FDT**	ICC: 0.92	ICC: 0.94 to 0.99	✖
**GaMA**	ICC: 0.75	p^b^<0.05	✖
**JHFT**	ICC: 0.84 to 0.97	ICC: 0.82 to 1.00	α: 0.95
**MMDT**	ICC: 0.79 to 0.87	✖	✖
**NHPT**	ICC: 0.92 to 0.95	ICC: 0.93 to 0.98	✖
**PHAM**	✖	✖	✖
**PPT**	ICC: 0.91	ICC: 0.91	✖
**RCRT**	✖	✖	✖
**SHAP**	ICC: 0.93	ICC: 0.89	✖
**SHFT**	ICC: 0.96 to 0.98^a^	ICC: 0.98	✖
**SODA**	r: 0.93	✖	α: 0.91
**T-MAP**	ICC: 0.93	✖	✖
**UBET**	ICC_[COT]_: ✖	ICC_[COT]_: 0.77 to 0.87	✖
	ICC_[MOU]_: 0.70 to 0.85	κ_[MOU]_: 0.68 to 0.82	
**UNBT**	ICC: 0.74 to 0.79	ICC: 0.72 to 0.73	α: 0.74
**WMFT**	ICC: 0.97^c^	ICC: 0.93 to 0.99^c^	α: 0.91^c^

Key: ICC, intraclass correlation coefficient, α = Cronbach’s alpha, r = Pearson Correlation Coefficient, κ = Cohen’s kappa, COT, completion of task; MOU, method of use.

^a^ Tested with pediatric patients with spastic hemiplegia.

^b^ *p*-value was used for a two and three factor RMANOVA.

^c^ Primarily tested with stroke patients.

**TABLE 7:** Validity scoring.

Test	Face	Content	Construct	External	Concurrent	Predictive
**ACMC**	Excellent	Adequate	Adequate	Excellent	Excellent	Adequate
**AHAP**	Adequate	Adequate	Adequate	Excellent	Excellent	Adequate
**AM-ULA**	Excellent	Excellent	Adequate	Excellent	Excellent	Excellent
**AOSDT**	Adequate	Adequate	No Evidence	Adequate	Excellent	Adequate
**ARAT**	Excellent	Adequate	Adequate	Excellent	Excellent	Adequate
**BAM-ULA**	Excellent	Adequate	Adequate	Excellent	Excellent	Adequate
**BBT**	Adequate	Poor	Adequate	Poor	Excellent	Poor
**CAPPFUL**	Excellent	Excellent	Adequate	Excellent	Excellent	Excellent
**CQT**	Excellent	Excellent	Excellent	Adequate	No Evidence	Excellent
**ECB**	Adequate	Adequate	No Evidence	Adequate	Excellent	Adequate
**FDT**	Adequate	Poor	Adequate	Poor	Excellent	Poor
**GaMA**	Adequate	Adequate	Adequate	Excellent	Adequate	Excellent
**JHFT**	Adequate	Adequate	Adequate	Adequate	Excellent	Poor
**MMDT**	Adequate	Poor	Poor	Poor	Excellent	Poor
**NHPT**	Adequate	Poor	Poor	Poor	Excellent	Poor
**PHAM**	Excellent	Excellent	No Evidence	Excellent	Excellent	Excellent
**PPT**	Adequate	Adequate	Adequate	Adequate	Excellent	Poor
**RCRT**	Adequate	Adequate	No Evidence	Poor	No Evidence	Poor
**SHAP**	Adequate	Adequate	Adequate	Excellent	Excellent	Excellent
**SHFT**	Adequate	Adequate	Adequate	Excellent	Excellent	Adequate
**SODA**	Adequate	Adequate	Adequate	Adequate	No Evidence	Adequate
**T-MAP**	Excellent	Excellent	Adequate	Excellent	Excellent	Excellent
**UBET**	Excellent	Excellent	Adequate	Excellent	Adequate	Excellent
**UNBT**	Excellent	Excellent	Adequate	Excellent	Excellent	Excellent
**WMFT**	Adequate	Adequate	Adequate	Adequate	Excellent	Adequate

### 3.1 Assessment of capacity for Myoelectric Control (Lindner et al., 2009; Lindner and Linacre, 2009; Burger et al., 2014; Lindner et al., 2014)

The Assessment of Capacity for Myoelectric Control (ACMC) is a versatile evaluation tool consisting of 30 items, measured by a rater who observes the user perform self-selected functional tasks. Designed for all ages and different levels of prosthetic limb use, each ACMC item is rated on a four-point scale from zero (incapable) to three (spontaneously capable). The ACMC tests multiple hand grasps using varied objects, in-hand manipulation, tool usage, and monitors progress without requiring the evaluator to have prior training. Although relatively comprehensive, the ACMC limitations include: no active mechanism to capture patient feedback, lengthy administration time (>30 min), and significant acquisition costs (∼$1000USD). The ACMC does demonstrate excellent test-retest and inter-rater reliability, with ICCs of 0.94 and 0.92–0.95 respectively, and performed at least adequately in all our validation scoring. However, its use with future multi-grasp prostheses may have limitations due to the score’s upper limit, and its specificity to myoelectric devices prevents the assessment of body-powered prostheses.

### 3.2 Anthropomorphic hand assessment protocol (Llop-Harillo et al., 2019)

The Anthropomorphic Hand Assessment Protocol (AHAP) is a specialized protocol designed to evaluate the grasping and retaining abilities of upper-limb prostheses. The AHAP comprises 26 tasks involving 25 common household items (spatula, chips can, key, etc.,.). The AHAP tests many grasp patterns and specifies which to use for each object. The grip patterns are: pulp pinch, lateral pinch, diagonal volar grip, cylindrical grip, extension grip, tripod pinch, spherical grip, and hook grip. Each task is individually scored for grasping and retaining by a rater using a 0, 0.5, or 1 rating, as dictated by the specific criteria provided in the AHAP paper appendix (Llop-Harillo et al., 2019). The AHAP performs strongly in many of our evaluation criteria due to its diverse range of grasp patterns, varying objects, and lack of prerequisite training. Nonetheless, the AHAP exhibits noteworthy limitations. Notably, scoring can be skewed due to the stringent guidelines for determining correct grasping. Additionally, it lacks an assessment of control systems, omits tasks involving tool usage, offers limited capability for tracking progress, lacks patient feedback, and necessitates a minimum of 30 min to complete. Despite these shortcomings, the AHAP exhibits excellent inter-rater reliability with an ICC of 0.969. Furthermore, the AHAP exhibits good test-retest reliability and internal consistency, as evidenced by an ICC of 0.839 and α of 0.846 respectively. The AHAP demonstrates adequate validity for evaluating the performance of current prostheses. Essentially, the AHAP is designed to assess the grasping capabilities of a prosthesis without factoring in control systems. While this serves as a useful measure in technical robotic contexts, its correlation with real-world prosthetic outcomes for patients remains uncertain.

### 3.3 Activities measure for Upper Limb amputees (Resnik et al., 2013a)

The Activities Measure for Upper Limb Amputees (AM-ULA) is a comprehensive, 18-item measure designed for adults with upper-limb amputation, which evaluates task completion, speed, movement quality, skillfulness of prosthetic use, and independence. This assessment is designed for adults and is compatible with all types of prosthetic devices. Scoring for the AM-ULA ranges from zero to 40, with higher scores denoting better functional performance. While the AM-ULA scored highly across most of our criteria with the only gap being patient feedback, it does pose significant challenges. The requirement for a certified hand therapist as a rater, coupled with lengthy administration (>30 min), makes the AM-ULA far less accessible. Consequently, inter-rater reliability has shown some variability, with ICCs ranging from 0.69 to 0.95. However, the test exhibits excellent test-retest reliability, with an ICC of 0.91, and strong internal consistency (α: 0.89–0.91). In terms of validity, the AM-ULA performs exceptionally well, earning ‘excellent' ratings in nearly all types. Overall, despite its strengths, the limited accessibility of the AM-ULA may restrict its widespread use.

### 3.4 Accessible, Open-Source Dexterity Test (Elangovan et al., 2022)

The Accessible, Open-Source Dexterity Test (AOSDT) is a novel assessment method designed to evaluate the performance of robots and adults with upper-limb amputation. The methodology includes 24 tasks using cylindrical and cuboidal objects conducted on a rotating platform. The tasks are divided into five distinct manipulation categories: simple manipulation of cylindrical/cuboidal objects, re-orientation of objects, fine manipulation of nuts and washers, tool tasks using screwdrivers to assemble/disassemble screws and nuts, and puzzle manipulation. The performance metrics of the AOSDT are derived from two parameters: the success rate and speed of task completion. Task success is measured on a 0–4 scale per specific criteria and the overall score is a weighted average of the scoring from each parameter (Elangovan et al., 2022). While the AOSDT scored well on most of our criteria, despite the lack of patient feedback, its recent development in 2022 caused it to lack extensive reliability testing. Although it appears valid for measuring the dexterity of different hand grasps, it does not evaluate control systems. Furthermore, a significant obstacle is its requirement for custom 3D printed parts and rotational test board. This time-consuming construction process and the current absence of comprehensive reliability testing may significantly hinder its adoption.

### 3.5 Action research arm Test (Shirley Ryan AbilityLab, 2016a; Physiopedia, 2023)

The Action Research Arm Test (ARAT) is a 19-item measurement tool designed for use by certified hand therapists to evaluate upper extremity and upper-limb prosthesis performance. The ARAT involves the completion of tasks grouped into four subscales: grasp, grip, pinch, and gross movement, with the performance of these tasks forming the individual’s score. Tasks are arranged in a descending order of difficulty, with the most complex task attempted first, based on the hierarchy suggested by Lyle et al., to enhance test efficiency (Physiopedia, 2023). Performance is rated on a four-point scale, with zero signifying no movement and three representing normal movement. While the ARAT scored well across most criteria, it does require a certified hand therapist to administer, lacks objects with varied compliances, excludes tool usage, has relatively high acquisition costs (∼$650USD), does not assess control systems, and lacks a method for patient feedback. However, the ARAT demonstrates excellent reliability, with ICCs of 0.965 and 0.998 for test-retest and inter-rater reliability, respectively. The ARAT also exhibits excellent internal consistency, with a α of 0.985, though this analysis was notably conducted with data from stroke patients rather than those with limb deficiencies. Overall, despite its strengths, the ARAT might be too time-consuming for a clinical setting, and could struggle supplying enough information for a research laboratory.

### 3.6 Brief activities measure for Upper Limb amputees (Resnik et al., 2018)

The Brief Activities Measure for Upper Limb Amputees (BAM-ULA) was developed as an alternative to the more comprehensive AM-ULA to address issues such as the lengthy completion time of approximately 30–35 min and a complicated scoring system that requires a trained clinician. The BAM-ULA streamlined this with a ten-item observational measure of activity performance, where each item is scored as either zero for ‘unable to complete' or one for ‘did complete'. The total score is derived from the sum of these individual item scores. The BAM-ULA demonstrated commendable reliability with a test-retest ICC of 0.91 and an internal consistency α of 0.83. Regarding validity, the BAM-ULA achieved at least an ‘adequate' rating in all categories. However, there are concerns that the simplicity and binary scoring system of the BAM-ULA may not adequately reflect the functional capabilities of the prosthesis and might fail to distinguish between the performances of more advanced prosthetic hands.

### 3.7 Box and Block Test (Shirley Ryan AbilityLab, 2012a)

The Box and Block Test (BBT) is a straightforward measure of dexterity and upper-extremity function, involving 150 wooden cubes, each 2.5 cm per side. The score is based on how many blocks a participant can individually transfer from one compartment, over a partition, to another within 60 s. Each successfully moved block earns a point. In terms of our criteria, the BBT scored relatively low due to its inability to measure different hand grasps or control systems, and its exclusive use of identical cubes as the objects with no variation or inclusion of tools. However, the BBT is straightforward to administer, quick, and its reliability has been thoroughly evaluated, scoring highly with a test-retest ICC of 0.96 and inter-rater ICC of 0.99. While the BBT has been validated in various contexts and is commonly chosen in clinical settings due to its time efficiency, it does not comprehensively capture the capabilities of current prostheses and will likely become increasingly outdated.

### 3.8 Capacity assessment of Prosthetic Performance for the Upper Limb (Kearns et al., 2018; Dynamics, 2023)

The Capacity Assessment of Prosthetic Performance for the Upper Limb (CAPPFUL) is an outcome measure tailored for adults with upper-limb deficiencies. This measure evaluates a user’s ability to perform 11 tasks (which require diverse hand grasp patterns to complete). It assesses across five distinct functional domains: control skills, component utilization, maladaptive/adaptive compensatory movements, and task completion. The CAPPFUL is also the only currently validated test that features a complementary patient feedback mechanism, developed to integrate with the task-based evaluation. However, there are challenges associated with its use. Specifically, it necessitates the involvement of an occupational therapist, specialized in upper-limb prostheses, who must also undergo additional training. Furthermore, its duration can be considered lengthy for clinical environments, averaging between 25–35 min. In terms of its psychometric attributes, CAPPFUL exhibits commendable reliability, with inter-rater reliability ICCs ranging from 0.88–0.99 and an internal consistency α of 0.79–0.82. The measure has also achieved ‘excellent' scores in the majority of validity categories. In summary, while the CAPPFUL stands out as a comprehensive assessment tool that includes patient feedback, its utility may be constrained by the specialized expertise required for its administration.

### 3.9 Carroll quantitative test of Upper Extremity Function (Carroll, 1965; Lu et al., 2011)

The Carroll Quantitative Test of Upper Extremity Function (CQT) was originally developed to assess hand function post-traumatic injury, but has been adapted for evaluating upper-limb prostheses. It involves 32 tasks using 18 objects, testing diverse actions to assess dexterity, arm motion, and, to some degree, strength. While some tasks necessitate a power grip, the test predominantly focuses on pinch positions, dedicating 16 tasks to assess the ability to pinch using the thumb in conjunction with each of the four fingers. Performance is scored from 0–3, based on observed task completion quality and is supplemented by a reading from a Smedley dynamometer. However, the CQT has limitations: mandates administration on a custom table, lacks a patient feedback component, has not been compared with other tests, and its reliability has only been verified in pediatric patients with spastic hemiplegia (Lu et al., 2011). Despite these challenges, the CQT excelled in our validation criteria, receiving scores of ‘excellent’ in the majority of validation types. While the CQT has overall good validity, its focus on pinch grips and lack of reliability testing may limit its adoption.

### 3.10 Elliott and Connolly Benchmark (Coulson et al., 2021)

The Elliott and Connolly Benchmark (ECB) is a dexterity evaluation tool designed for use with robotic hands along with upper-limb prostheses. It involves eight objects and employs 13 manipulation patterns including pinch, dynamic tripod, squeeze, twiddle, rock, rock II, radial roll, index roll, full roll, rotary step, interdigital step, linear step, and palmar slide. The ECB initially scores performance on a binary basis—success or failure—based on the specific criteria for each pattern. Following this, it uses custom quantitative metrics to track the translations and rotations of each object along a specified hand coordinate axis (Coulson et al., 2021). The ECB performed well against our criteria, falling short only in tool usage, capability to monitor patient improvement, and providing a method for patient feedback. Furthermore, while the ECB does not explicitly require prerequisite qualifications, the calculations require extensive mathematical knowledge that likely necessitates training or a researcher to perform the test. Furthermore, its creation in 2020 has limited extensive reliability testing. Although the ECB performed satisfactorily according to our validity criteria, its lack of reliability testing suggests that more comprehensive testing may be needed before it can be considered for widespread use.

### 3.11 Functional dexterity Test (Shirley Ryan AbilityLab, 2017)

The Functional Dexterity Test (FDT) is primarily intended to evaluate the functionality of the three-jaw chuck grasp pattern in individuals with hand injuries, but it has also been applied in the assessment of upper-limb prostheses. The test setup features a 16-peg board placed 10 cm from the edge of a table. Participants must pick up each peg, flip it over, and reinsert it following a zig-zag trajectory. The FDT’s scoring system considers both net time (the actual time taken to complete the test) and total time (net time plus any penalty seconds). Most errors incur a 5 s penalty, while dropping a peg results in a 10 s penalty. A total time exceeding 55 s suggests a non-functional hand. The FDT scored relatively low against our criteria, as it only assesses one hand grasp, lacks a control system evaluation, does not evaluate tool usage, and lacks patient feedback. However, it does assess in-hand manipulation, and is quick and straightforward to conduct. Extensive reliability testing shows an excellent test-retest ICC of 0.92 and inter-rater ICC of 0.94:0.99. Despite this, our validity assessment found the FDT to be moderately poor as it fails to comprehensively evaluate current prostheses. While the FDT is a common choice clinically due to its simplicity and quick administration, it likely lacks the capability for a comprehensive assessment of current and future prostheses.

### 3.12 Gaze and Movement Assessment (Gaze and Movement Assessment GaMA, 2019; Williams et al., 2019; Marasco et al., 2021)

The Gaze and Movement Assessment (GaMA) is an innovative tool for evaluating upper-limb prostheses, utilizing motion capture and eye tracking for functional tasks. It provides insights into hand-eye coordination and overall movement quality. While the GaMA does not directly assess multi-grasp dexterity, it reveals the influence of different grasps on movement kinematics. Furthermore, it is highly sensitive in detecting functional changes across control systems and prosthetic components. One significant limitation is its demanding setup, with equipment costs beginning at $2000USD, coupled with the need for specialized expertise for optimal use. The GaMA does feature good reliability, with a reported test-retest reliability ICC of 0.75 along with a RM-ANOVA determining its inter-rater reliability to be strong at a 95% confidence level. In terms of validity, the GaMA’s detailed analysis of movement kinematics and compensatory actions suggests robust predictive validity, positioning it as an invaluable tool for studying advanced prostheses in the future. However, its setup, duration, absence of patient feedback, intricate analysis, and cost might make it more suitable for research rather than routine clinical use.

### 3.13 Jebsen hand function Test (Shirley Ryan AbilityLab, 2012b; Panuccio et al., 2021; Sığırtmaç and Öksüz, 2021)

The Jebsen Hand Function Test (JHFT) is primarily used to evaluate the speed of upper-limb function. It includes seven subtests: writing, card-turning, moving small objects, stacking checkers, simulated feeding, moving light objects, and moving heavy objects. Each subtest measures the time taken in seconds to complete each task, beginning with the non-dominant hand, followed by the dominant hand. The JHFT scored well in our criteria, lacking only in the evaluation of objects with varying compliances and a method for patient feedback. Extensively tested for reliability, the JHFT is very reliable, demonstrating a test-retest ICC ranging from 0.84 to 0.97, an inter-rater ICC of 0.82–1.00, and an internal consistency α of 0.95. While the JHFT scored fairly well in our validity testing, it is limited by high acquisition costs (∼$400USD) and exclusively measuring speed. Consequently, the JHFT might be insufficient in delivering comprehensive data for research laboratories.

### 3.14 Minnesota manual dexterity Test (Desrosiers et al., 1997)

The Minnesota Manual Dexterity Test (MMDT) is an evaluation tool designed to measure hand-eye coordination and arm-hand dexterity, primarily focusing on gross motor skills. The MMDT consists of two-timed subtests: placing and turning. In the placing test, a participant is required to move 60 disks located above the testing board into the board’s 60 corresponding cutouts. The turning test begins with the disks already placed in the board’s cutouts. Participants must pick up each disk with their left hand, pass it to their right, flip it, and reinsert it into its original hole, following a zig-zag pattern. This process is then repeated in reverse, with the right hand passing the disk to the left hand. The MMDT’s scores are based on the speed of completion, not the quality of task performance. The MMDT also does not vary hand grasps, measure control systems, use tools, and is relatively expensive (∼$350USD). However, the MMDT does assess in-hand manipulation and shows good test-retest reliability with an ICC range of 0.79–0.87. Despite its quick evaluation time and good reliability, the MMDT might be limited in comprehensively evaluating all aspects of current or future prostheses.

### 3.15 Nine-hole peg Test (Shirley Ryan AbilityLab, 2022)

The Nine-Hole Peg Test (NHPT) is a functional assessment tool initially created to measure an impaired hand’s speed of motor function. However, it has been adapted for use with upper-limb prostheses. The test requires participants to individually pick up nine pegs from a container and place them into corresponding holes on a board, with scoring dependent on completion speed (it does not consider quality of task execution). An alternative scoring method is provided, which consists of the amount of pegs a participant can place within a designated time limit, typically 50 or 100 s. Despite its simplicity, speed, and high reliability—evidenced by a test-retest ICC of 0.92–0.95, and an inter-rater ICC of 0.93 to 0.98—the NHPT underperforms against our evaluation criteria. It only assesses in-hand manipulation over time, neglecting multiple grasp patterns, control system assessment, manipulation of diverse objects, tool usage, or patient feedback. In terms of validity, the NHPT performed poorly, given its inability to comprehensively evaluate the functionalities of hand prostheses. While the NHPT is time efficient and therefore a common choice clinically, it is likely unable to comprehensively evaluate the dexterity and control system capabilities of modern prostheses.

### 3.16 Prosthetic hand assessment measure (Hunt et al., 2017)

The Prosthetic Hand Assessment Measure (PHAM) quantifies traditionally qualitative performance metrics for upper-limb prostheses. Using a custom PVC frame with four LED-marked sections, it assesses prosthetic functionality at various arm angles. Participants move one of four basic objects (e.g., a cylinder representing a glass) between sections, depending on activated LEDs, using grip patterns like power, tripod, pinch, or key. The PHAM employs five inertial measurement units and a custom piezoresistive mat to record movement details, which are then analyzed using custom equations to assess metrics like 3D deviations in the chest and shoulder, 2D translational displacement, and completion rate (Hunt et al., 2017). As for its psychometric properties, the PHAM has yet to undergo reliability testing, though it achieved mostly ‘excellent' scores in our validation criteria. Though comprehensive, the PHAM’s reliance on specialized equipment, high cost (∼$500USD), intricate administration, and scoring, coupled with its untested reliability, might limit its broader adoption.

### 3.17 Purdue pegboard (Tiffin and Asher, 1948; Shirley Ryan AbilityLab, 2013a; Lindstrom-Hazel and VanderVlies Veenstra, 2015)

The Purdue Pegboard Test (PPT) is an evaluation measure designed to measure gross movements of fingers and finger dexterity. The board used in the test features four cups at the top and two vertical rows of 25 small holes down the center. The two outer cups hold 25 pins each, the cup to the immediate left contains 40 washers, and the one to the right of the center holds 20 collars. The test starts with the participant using their right hand to insert as many pins as they can into the right row within 30 s, followed by the left hand placing pins into the left row for 30 s. Subsequently, both hands insert pins into both rows within another 30 s period. The final task requires the participant to assemble as many pins with washers and collars as they can using both hands within 60 s. The test produces five scores: the number of pins inserted by the right hand, the left hand, both hands, the sum of these three, and the number of assembled pins. In our evaluation, the PPT received a moderate score. While it is exceptional at assessing finger dexterity and indirectly measures multiple grasp patterns, the PPT does not directly evaluate control systems, tool usage, or provide a method for patient feedback. It does show excellent test-retest and inter-rater reliability, with ICCs of 0.91 for both. In terms of validity, while the PPT effectively evaluates finger dexterity and in-hand manipulation, it falls short in providing a comprehensive assessment of prostheses dexterity and control. However, the PPT is likely a good evaluation tool for clinicians who wish to effectively evaluate finger dexterity.

### 3.18 Refined clothespin relocation Test (Hussaini and Kyberd, 2017; Hussaini et al., 2019)

The Refined Clothespin Relocation Test (RCRT) was developed to evaluate individuals’ proficiencies in using a prosthesis, specifically their compensatory movements and the time taken to perform a grasping and repositioning task. Initially researched in a motion capture laboratory, the RCRT has been adapted for clinical application (Hussaini et al., 2019). The test requires patients to relocate three clothespins from a horizontal plane to three different locations on a vertical pole set at low, medium, and high levels. Unlike traditional tests, the RCRT does not use a conventional scoring system. Instead, it compares the performance of prosthesis users to a control group of able-bodied individuals. Despite its simplicity and its ability to assess in-hand manipulation and the path of motion for clothespin relocation, the RCRT performed poorly against our evaluation criteria. It does not evaluate multiple grasp patterns, control systems, varying objects, tool usage, or offer a method for patient feedback. Furthermore, despite being established in 2016, the RCRT has no published reliability testing. For our validity assessment, the RCRT falls short in providing a comprehensive evaluation of current or future prostheses. Due to its limitations and the lack of robust reliability data, the RCRT may face limited use in clinical and research applications.

### 3.19 Southampton hand assessment procedure (Adams et al., 2009; SHAP, 2023)

The Southampton Hand Assessment Procedure (SHAP) is specifically designed to evaluate the functionality and efficiency of upper-limb prostheses, comprising six abstract objects and 14 Activities of Daily Living (ADL) tasks. All tasks are timed by the individual taking the test in an attempt to reduce the reliance on the observer or clinician’s reaction times. The objects, placed on a dual-sided board with a blue felt side for abstract tasks and a red plastic side for ADL tasks, are timed and recorded by the assessor. These timings are then normalized to a score of 100 using a method devised by Light, Chappell, & Kyberd (SHAP, 2023). The scoring software, available for purchase through the SHAP website, associates each of the 26 tasks with one of six prehensile patterns, enabling the creation of a SHAP Functionality Profile—a numerical assessment of hand function highlighting areas of extraordinary skill or potential impairment. Notably, SHAP scores can exceed 100 for exceptionally quick task completion, while scores under 100 may indicate functional impairment. While the SHAP performed well in our evaluation, it lacks a method for patient feedback, and has two significant drawbacks: a trial takes at least 45 min to complete, and it costs over $2500USD for the equipment and license to the proprietary scoring software. However, with ICC values of 0.93 and 0.89 for test-retest and inter-rater reliability, respectively, the SHAP is considered very reliable. It also performed well in our validity assessment, indicating that it can accurately measure the dexterity of current and future prostheses. However, due to its lengthy administration time, absence of control system assessment, and high cost, its clinical and research use will likely be limited.

### 3.20 Sollerman hand function Test (Sollerman and Ejeskär, 1995; Shirley Ryan AbilityLab, 2013b; Ekstrand et al., 2016)

The Sollerman Hand Function Test (SHFT) is an evaluative tool designed to measure the functionality of adult hands impaired due to injury or disease, but has been adapted for upper-limb prosthesis evaluations. It assesses the performance of seven distinct hand grips: pulp pinch, lateral pinch, tripod pinch, five-finger pinch, diagonal volar grip, transverse volar grip, spherical volar grip, and extension grip. The SHFT consists of 20 items, each encompassing 20 subtasks, each of which is rated on a scale of zero to four. Scores are determined based on the time taken to complete the task, the quality of task execution, and the assessor’s perception of the task’s difficulty. The SHFT scored highly in our evaluation criteria, only lacking a control system assessment and a method for patient feedback. The SHFT also boasts excellent reliability, with a test-retest ICC of 0.96:0.98 and an inter-rater ICC of 0.98. However, it is important to note that the test-retest reliability was predominantly evaluated on stroke patients (Ekstrand et al., 2016). In our validity assessment, we ascertain that the SHFT can accurately evaluate the dexterity of various hand grasps with current and future prostheses. The SHFT may be a good test for dexterity in a research environment, but would benefit from a reliability evaluation specifically with prostheses, the inclusion of a patient questionnaire, and an incorporated assessment of control systems.

### 3.21 Sequential occupational dexterity assessment (Van Lankveld et al., 1996; Liu et al., 2016)

The Sequential Occupational Therapy Dexterity Assessment (SODA) is an assessment tool originally designed to evaluate hand function in individuals with rheumatoid arthritis. However, it has been adapted for use with upper-limb prosthetics. The SODA consists of 12 tasks, each rated from zero to four based on performance and zero to two based on perceived difficulty, contributing to a total evaluation score. A significant challenge in adapting the SODA for prosthetic users is that six tasks necessitate the use of both hands, yet only one hand is scored. This approach is predicated on the presumption that rheumatoid arthritis would symmetrically affect both hands, thereby yielding identical scores. However, this may not be applicable to the vast majority of people with limb deficiencies, who have a single prosthetic limb. The SODA met most of our evaluation criteria, except for control system assessment, tool usage, needing at least 30 min to administer, and a method for patient feedback. The reliability of the SODA remains a point of contention in our view. It has an internal consistency α of 0.91, but the test-retest reliability was assessed using the Pearson correlation coefficient (r) instead of the ICC and lacks any testing for inter-rater reliability. While the SODA did receive an r value of 0.93, the ICC value would offer a more accurate representation of reliability because it accounts for the difference of the means of measures (Liu et al., 2016). As for validity, the SODA shows promise but requires further adaptation for prosthetics before it can be used clinically.

### 3.22 Timed measure of activity performance in persons with Upper Limb Amputation (Resnik et al., 2017b)

The Timed Activity Performance in Persons with Upper Limb Amputation (T-MAP) was developed to provide a timed measure for the functional outcomes of persons with upper-limb amputation. Although the T-MAP can also assess performance without a prosthesis, this aspect was not considered in our review. The T-MAP incorporates five tasks: drinking water, face washing, food preparation, eating, and dressing. Therapists assess both the time taken and the level of independence displayed during each activity. The independence metric uses a 3-point scale: 1 indicating dependency, 2 for verbal assistance required, and 3 for independent action, with or without aid. By aggregating the independence ratings and times, overall scores for both parameters are derived. In our evaluation, the T-MAP performed exceptionally well, only lacking a patient feedback mechanism. It boasts a test-retest reliability ICC of 0.93, but lacks inter-rater reliability data. On most validity aspects, the T-MAP achieved an ‘Excellent' rating. Overall, while the T-MAP provides insights into the time it takes for someone with a limb deficiency to perform daily activities, its sole focus on timing limits its depth of analysis.

### 3.23 Unilateral below elbow Test (Bagley et al., 2006)

The Unilateral Below Elbow Test (UBET) evaluates bimanual activities in both prosthesis wearers and non-wearers (for this review, we will be excluding the non-wearers portion). The UBET employs four age-specific categories, reflecting the developmental stages of hand function (2–4, 5–7, 8–10, and 11–21). Each category contains nine tasks, utilizing everyday household objects relevant to that age’s hand development level. UBET’s dual rating system, ‘Completion of Task' and ‘Method of Use', allows for assessing the overall task completion quality and recognizing functional disparities among different control systems. However, UBET’s limitations include the need for an occupational therapist during administration, its lengthy procedure, an age ceiling of 21, and the absence of integrated patient feedback. In terms of reliability, the ‘Completion of Task' has an inter-rater ICC ranging from 0.77 to 0.87, while ‘Method of Use' shows a test-retest reliability ICC between 0.70 and 0.85, and a good Cohen’s kappa value (equal relevance to an ICC) of 0.68–0.82 for inter-rater reliability. In validity metrics, the UBET predominantly scores as ‘excellent'. Overall, the UBET serves as a useful tool for pediatric research applications.

### 3.24 University of New Brunswick Test of prosthetics function (Resnik et al., 2013b; Burger et al., 2014)

The University of New Brunswick Test (UNBT) is an evaluative methodology created to measure upper-limb prosthetic function in those with limb loss aged two to 21. The UNBT, capable of evaluating both body-powered and myoelectric prostheses, categorizes participants into four age groups: 2–4, 5–7, 8–12, and 13–21. All age groups include three subtests featuring ten tasks each. These tasks are assessed on two aspects: spontaneity and skill of prosthetic function. Both elements are scored on a 0–4 scale, and the scores for each category are tallied at the end of each subtest. The UNBT also provides a therapy recommendation chart based on the scores obtained in each category. In our evaluation criteria, the UNBT performs exceptionally well, falling short only in the relatively high cost of $500, absence of a questionnaire—likely due to patient age—, and a rather lengthy administration time of at least 30 min. The UNBT also demonstrates good reliability, with a test-retest ICC of 0.74:0.79, an inter-rater ICC of 0.72:0.73, and an internal consistency α of 0.74. In terms of validity, the UNBT is among the most comprehensive tests we reviewed for evaluating the dexterity and control systems of modern and future prostheses. The UNBT may be a great option for use in a research laboratory but is likely too long to feasibly administer in a clinical setting.

### 3.25 Wolf motor function Test (Shirley Ryan AbilityLab, 2016b)

The Wolf Motor Function Test (WMFT) is a diagnostic tool initially designed for assessing upper-limb dexterity and strength in stroke recovery patients, but it has been adapted for use with prostheses. The WMFT, originally consisting of 21 items and tasks, is now typically used with 17 items and tasks, with each task capped at a maximum of 120 s. The first six objects are used for timed functional tasks; items seven through fourteen are used to measure strength, and the remaining objects assess movement quality. Each item is scored on a scale of zero to five, and the final score is the aggregate of all item scores. In our evaluation criteria, the WMFT scored well, falling short only in control system assessment, the absence of a patient feedback questionnaire, and a relatively long administration time of at least 30 min. Although the WMFT demonstrates excellent reliability results, primarily with stroke patients, with a test-retest ICC of 0.97, an inter-rater ICC of 0.92:0.99, and an internal consistency α of 0.91, it would benefit from further reliability testing with prostheses. Our validity assessment also gave the WMFT high marks. However, its administration time may restrict clinical use and it should likely incorporate a control systems assessment, along with updated reliability testing, before widespread implementation could occur in a research setting.

## 4 Discussion

In the current state of upper-limb prosthetic research, a significant discrepancy exists between the pace of mechatronic development and the available evaluation methodologies. Among the reviewed tests, there is a limited ability to comprehensively measure the performance of upper-limb prostheses in terms of directly quantifying both multi-grasp dexterity along with the impact of varying control systems. This highlights the need for urgent action to establish standardized, comprehensive evaluation methodologies suitable for both clinical and research settings. However, addressing this issue is a nuanced challenge since the success of a test can often be influenced by competing interests. Clinical environments, typically overseen by physical therapists, occupational therapists, and certified hand therapists, often face tight schedules that blend evaluation with treatment. Here, quick tests like the BBT, MMDT, and NHPT are likely preferred. Yet, their current forms inadequately capture the capabilities of modern prostheses, particularly in multi-grasp dexterity. Conversely, research laboratories tend to lean toward exhaustive tests like the AHAP, AM-ULA, SHAP, and UNBT. However, these tests’ extensive setups, niche objects, significant costs, and intricate procedures have hampered widespread implementation. Therefore, while validated tests are available, their limitations have resulted in inconsistent adoption that has also hindered a unified and standardized evaluation framework. This lack of standardization and validated evaluation measures has caused multiple problematic consequences. Notably, new prosthetic devices tend to be assessed using methods devised by their own developers, introducing potential bias and undermining validity. Furthermore, the lack of a unified, standard assessment process has resulted in redundancy among current validated tests, further complicating the evaluation process. One example of this was demonstrated by Burger et al., who found that the ACMC and UNBT are equally capable of evaluating myoelectric prostheses (Burger et al., 2014).

In light of these challenges, we are advocating for a dual testing approach. While clinical and research settings should both value certain aspects, such as affordability and simple administration, each approach needs to consider and accommodate their differing constraints and objectives. Clinically, a swift, user-friendly test should be used, primarily focused on tracking patient progress while still considering multi-grasp dexterity and the effect of different control systems. For research settings, a more rigorous test that ensures a comprehensive analysis of the prosthesis’s capabilities is essential. However, we also believe that both versions should maintain a degree of commonality. This allows clinical results to be compared with those of the more detailed research assessment. This will promote improved communication between clinicians and researchers along with ensuring both parties have access to the most consistent, relevant, and insightful data.

An essential component lacking in 24 of the 25 previously mentioned validated tests is an integrated mechanism to gather patient feedback. We maintain that patient feedback, often collected *via* questionnaires and surveys, forms a crucial part of a comprehensive upper-limb prosthesis evaluation (Virginia Wright et al., 2001; Wright et al., 2003). Currently, self-reported surveys and task-based assessments are designed independently, leaving assessors to combine and interpret data from separate, potentially incompatible sources. Research, such as the study by Burger et al., has underscored the importance of questionnaires for yielding invaluable insights and revealed the limitations of exclusively depending on clinical tests (Burger et al., 2004). There are deeper underlying predictors of performance, often first identified by patients, that extend beyond just the quality of the prosthesis or its control system. Factors such as socket fit, heat or sweat management, skin irritation, suspension or harnessing, and overall discomfort not only impact an individual’s ability to use and effectively operate their prosthesis but also determine its consistent use (if it is not comfortable, the prosthesis will not be used) (Burger et al., 2004; Smail et al., 2021; Salminger et al., 2022). Furthermore, the psychosocial impact of a prosthetic device on a person’s self-image, confidence, and social interactions can only be assessed through patient feedback, informing design improvements and support services (Armstrong et al., 2019; Roșca et al., 2021; Smail et al., 2021). We believe patient feedback is indispensable in evaluating the prosthesis’s overall effectiveness, providing a holistic view that not only considers the physical and mechanical aspects but also addresses the psychological and social implications.

## 5 Conclusion

This narrative review assessed upper-limb prosthetic dexterity and control system assessment techniques. Our primary objective was to analyze the essential characteristics and psychometric attributes of these evaluations and identify any existing gaps in the field. Additionally, we aimed to provide a resource to assist clinicians and researchers in selecting appropriate tools for assessing dexterity and control systems. Our analysis indicated commonly used clinical assessments, primarily chosen by time constraints, are limited and often fail to capture the dexterity and control system capabilities of modern prostheses. Conversely, current comprehensive tests may be suitable for certain research labs; however, their lengthy administration, significant cost, and complexity may hinder widespread adoption. Therefore, we propose a dual testing approach: clinicians should use quick, efficient tests for patient progress, while research labs need detailed tests for prosthesis capabilities. Both tests, however, should be cost-effective and user-friendly for evaluators and participants in an effort to help standardize the testing process. We also believe patient feedback needs to be included since it is indispensable in evaluating the prosthesis’s overall effectiveness, providing a holistic view that not only considers the physical and mechanical aspects but also addresses the psychological and social implications. Thus, both clinical and research environments urgently require the creation or refinement of tests to more accurately evaluate the dexterity and control systems of modern upper-limb prosthetics.
